# Finite Element Study on the Preservation of Normal Knee Kinematics with Respect to the Prosthetic Design in Patient-Specific Medial Unicompartmental Knee Arthroplasty

**DOI:** 10.1155/2020/1829385

**Published:** 2020-03-18

**Authors:** Yong-Gon Koh, Kyoung-Mi Park, Kyoung-Tak Kang

**Affiliations:** ^1^Joint Reconstruction Center, Department of Orthopaedic Surgery, Yonsei Sarang Hospital, 10 Hyoryeong-ro, Seocho-gu, Seoul 06698, Republic of Korea; ^2^Department of Mechanical Engineering, Yonsei University, 50 Yonsei-ro, Seodaemun-gu, Seoul 03722, Republic of Korea

## Abstract

Alterations in native knee kinematics in medial unicompartmental knee arthroplasty (UKA) are caused by the nonanatomic articular surface of conventional implants. Technology for an anatomy mimetic patient-specific (PS) UKA has been introduced. However, there have been no studies on evaluating the preservation of native knee kinematics with respect to different prosthetic designs in PS UKA. The purpose of this study was to evaluate the preservation of native knee kinematics with respect to different UKA designs using a computational simulation. We evaluated three different UKA designs: a nonconforming design, an anatomy mimetic design, and a conforming design for use under gait and squat loading conditions. The results show that the anatomy mimetic UKA design achieves closer kinematics to those of a native knee compared to the other two UKA designs under such conditions. The anatomy memetic UKA design exhibited a 0.39 mm and 0.36° decrease in the translation and rotation, respectively, in the swing phase compared with those of the natural knee. In addition, under the gait and squat loading conditions, the conforming UKA design shows limited kinematics compared to the nonconforming UKA design. Our results show that the conformity of each component in PS UKA is an important factor in knee joint kinematics; however, the anatomy mimetic UKA design cannot restore perfect native kinematics.

## 1. Introduction

Unicompartmental knee arthroplasty (UKA) in patients with isolated medial osteoarthritis (OA) in a knee joint is known to be a standard procedure leading to good postoperative results [1]. Reliable clinical results during the first decade of use encouraged surgeons to expand the application of UKA to younger and more active patients [2, 3]. There are several main advantages of UKA compared to total knee arthroplasty (TKA). For example, the postoperative range of motion is better, patients feel more normality in their motion, and the prevalence of postoperative complications, such as deep venous thrombosis, pulmonary embolism, and infection, are lower [4–6]. Furthermore, UKA has the benefit of both preserving the cruciate ligaments and delivering normal-like kinematics compared to the TKA [7]. In terms of survivorship, the results of UKA at 10 years have become competitive to those reported for TKA [8].

Traditionally, UKA has been available in a limited number of sizes leading to a debate on poor patient outcomes when the implant sizing is a contributing factor [9]. Numerous anatomical studies have shown a wide range of variability in the size and shape of the medial and lateral tibial baseplates [10–12]. To overcome such a problem, patient-specific (PS) implants have been recently introduced for UKA patients [10]. Customized UKA offers PS femoral and tibial prostheses, as well as PS instrumentation for cutting bones [9].

Carpenter et al. reported that significantly less cortical rim overhang and undercoverage have been observed when applying PS UKA [9]. Therefore, they claimed that a PS prosthesis provides a superior cortical bone coverage and fit, while minimizing the overhang and undercoverage found in an off-the-shelf prosthesis [9]. Koeck et al. showed that PS UKA can restore the leg axis reliably, provide a medial proximal tibial angle of 90°, avoid the malpositioning of an implant, and ensure maximal tibial coverage [13]. In addition, Steklov et al. investigated the use of a novel prosthetic design utilizing a PS sagittal J-curve on the femoral component combined with a novel constant and patient-derived femoral coronal curvature, and to assess the tibiofemoral (TF) contact area and contact stress on a femur matched curved tibial insert [14]. They showed that a novel approach combines the unique benefits of the PS geometry with validated design concepts for minimizing wear [14]. However, it misses an important point in that the medial and lateral tibial plateaus have asymmetric geometries with a slightly dished medial and a convex lateral plateau in the native knee [15]. The biomechanics of the medial and lateral menisci are substantially different [16, 17]. However, there have been no studies on evaluating the conformity design of a tibial insert in PS UKA.

The aim of this study was to compare the kinematics on a PS femoral component with a conforming tibial insert with respect to the different designs. We categorized the PS medial UKA designs into three groups with respect to their conformity: nonconforming- (NC-) PS UKA, anatomy mimetic (AM)-PS UKA, and conforming- (C-) PS UKA. We hypothesized that AM-PS UKA provided kinematics closer to those of the normal knee joint.

## 2. Materials and Methods

### 2.1. Development of PS UKA

**Figure 1:**
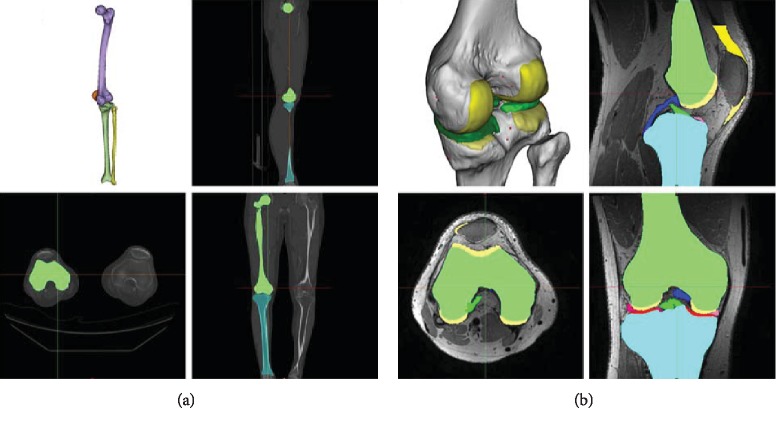
3D medical imaging data of (a) CT and (b) MRI used in the development of the PS UKA models.

The PS medial UKA was based on previously existing three-dimensional (3D) knee joint model [18–22]. 3D knee joint model was developed using medical imaging data. The model was developed using computed tomography (CT) and magnetic resonance imaging (MRI). The image data were imported into Mimics version 14.1 (Materialise, Leuven, Belgium) for editing and 3D reconstruction (Figure 1). The PS UKA design was conducted using Unigraphics NX (Version 7.0, Siemens PLM Software, Torrance, CA, USA).

**Figure 2:**
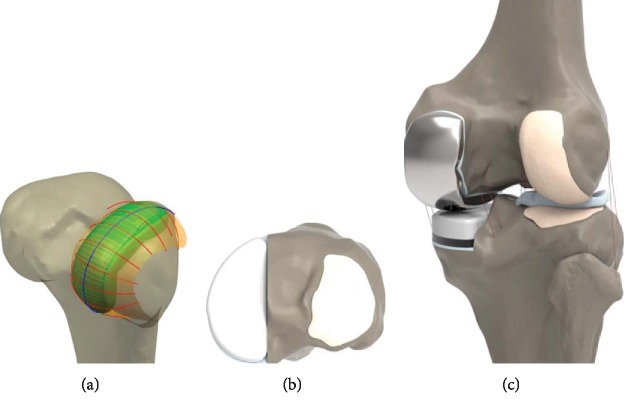
Design of the PS UKA: (a) femoral component using subject's anatomic curves in sagittal and coronal planes; (b) tibial component; (c) validated FE model with PS UKA.

Planes were introduced through the intersection of condyles in both the sagittal and coronal views. [14, 18, 23–25]. Intersection curves were used to extract the articulating surface geometry in sagittal and coronal plane. Sagittal geometry of the femoral component was respected to patient's bone, and the resultant sagittal implant radii vary along the anteroposterior dimension of the implant [14, 18, 23–25]. The coronal curvatures of the patient are measured at multiple positions along the length of the femoral condyle (Figure 2). The tibial component is designed based on the patient's tibia 3D model to ensure complete cortical rim coverage. Implant is made to be PS, it provides the potential for complete cortical rim coverage that cannot be achieved with a conventional implant [26].

**Figure 3:**
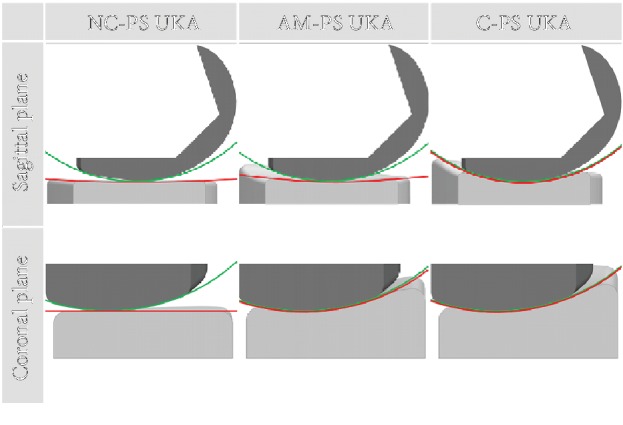
Cross section of the PS-TKA model according to three kinds of conformity by applying the curvature radius ratio in the sagittal and coronal plane.

We designed three different tibial insert conformities in Figure 3 [21]. NC-PS UKA developed flat design as the initial conforming tibial insert. A flat tibial insert was used for patient-specific fixed-bearing UKA [21, 27]. AM-PS UKA was developed second of tibial insert design applied the real medial geometry [21]. Femoral component coronal curvature varies and edge loading may occur in the conforming design, but various tibial insert designs can be applied in PS UKA. Therefore, the third design is C-PS UKA [21]. Femoral component designs were all same in PS UKA [21].

### 2.2. Finite Element Model

PS UKA design was also used in the development of the finite element (FE) model. The intact model was previously developed and validated [18–22].

The FE model comprises the TF and patellofemoral (PF) joints and major ligaments (Figure 2). The ligaments insertion points were set with respect to the anatomy obtained from the magnetic resonance imaging sets of the subject and descriptions provided in previous studies [28–30]. All ligaments were modeled as nonlinear springs in the previous study [21, 31, 32]. The ligaments were simulated as nonlinear force elements in the previous study [21]. The bony structures were modeled as rigid bodies using four-node shell elements [21, 33]. The interfaces between the articular cartilage and the bones were modeled as fully bonded [21, 33]. Six pairs of TF contact between the femoral cartilage and the meniscus, the meniscus and the tibial cartilage, and the femoral cartilage and the tibial cartilage were modeled for both the medial and lateral sides of the joint [18, 21]. The heights of the tibial insert for three different designs were matched to the original bone anatomy using a sagittal cross-sectional image, then aligned with the mechanical axis, and positioned at the medial edge with a square (0°) inclination in the coronal plane of the tibia [18, 21, 34]. The rotating alignment was defined as the line parallel to the lateral edge of the tibial baseplate passing the center of the femoral component fixation peg. PS UKA implanted model applied a 1 mm cement gap between the component and bone. The femoral component, PE insert, and tibial baseplate materials were cobalt–chromium alloy (CoCr), UHMWPE, and titanium alloy (Ti6Al4V), respectively. According to previously published data, the material properties, in terms of Young's Modulus (E) and Poisson's Ratio, are as follows: CoCr: *E* = 195 GPa, *ν* = 0.3; UHMWPE: *E* = 0.94 GPa, *ν* = 0.46; and Ti6Al4V: *E* = 110 GPa, *ν* = 0.3 [34–36]. Solid modelling and meshing were performed by using Hypermesh 11.0 (Altair Engineering, Inc., Troy, Michigan). Convergence was defined to be a relative change of more than 5% between two adjacent meshes with a mean edge length of 1.2 mm. The femoral component has contact with the tibial insert. The coefficient of friction between the femoral component and tibial insert was chosen as 0.04 [36].

This FE investigation included the three types of loading conditions corresponding to the loads used in the experiment for a model validation and predictions for daily activity loading scenarios in the previous study [21, 22, 33]. Under the first loading condition, 150 N was applied to the tibia with 30° and 90° flexion in the FE knee joint in order to measure the anterior-posterior (AP) tibial translations [21, 22]. Additionally, a second axial loading of 1150 N was applied to the model in order to obtain the contact stresses and compare them to those reported in a published FE knee joint study [21, 22, 33]. The third loading condition corresponding to the gait cycle and squat loading was applied to evaluate the knee joint mechanics [21, 22]. A computational analysis was conducted using an AP force applied to the femur with respect to the compressive load applied to the hip with a constrained femoral internal-external (IE) rotation, free medial-lateral translation, and knee flexion determined through a combination of the vertical hip and the load of the quadriceps. Thus, a six degree-of-freedom TF joint was created [37–39]. A proportional-integral-derivative controller was incorporated into the computational model in order to control the quadriceps in a manner similar to that used in a previous experiment [40, 41]. A control system was used to calculate the instantaneous displacement of the quadriceps muscle, which was required to match the same target flexion profile as that in the experiment. IE and varus-valgus torques were applied to the tibia, while the remaining tibial degrees of freedom were constrained [37–39].

The FE model was analyzed using the ABAQUS software (version 6.11; Simulia, Providence, RI, USA). This study investigated and compared the kinematics of PS UKA designs with three different conformities from a native knee. In addition, the previous study, Kinematics of the PS mobile-bearing UKA designs, was compared [22]. Kinematics of PS USA designs were calculated based on Grood and Suntay's definition of a joint coordinate system [42].

## 3. Results

The intact model was compared with the experimental results using FEM simulation in the previous study, and proved its validity by showing results similar to literature [22].

The kinematics in PS UKA designs with three different conformities were compared with the validated normal knee under gait cycle and squat loading conditions. In addition, the results of the previous study, the design of the PS mobile-bearing UKAs, were compared. The mobile-bearing UKA designs are three types. The first is the flat design for a mobile bearing (FMB), second is the tibial plateau anatomy mimetic design for a mobile-bearing AMB), and third is the conformity-increasing design for a mobile bearing (CMB) [22].

**Figure 4:**
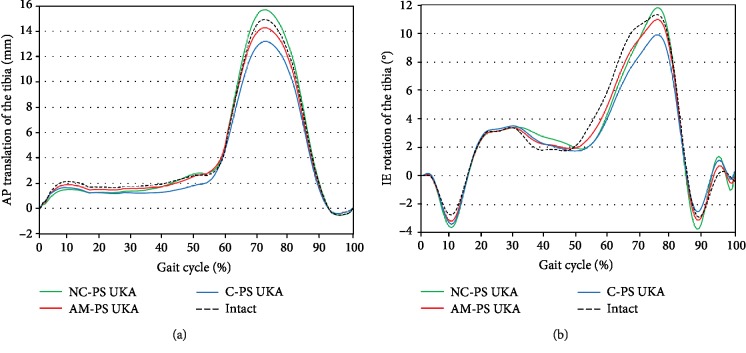
Comparison of (a) AP translation and (b) IE rotation of the tibia for the three types of PS UKA and natural knee model under the gait cycle.

AP translations and IE rotations of the tibia for the three types of PS UKA and natural knee under the gait cycle are shown in Figure 4. AP translations of the tibia in the three PS UKA models were very similar to those predicted for a native knee with 0.4 mm maximum deviation during the stance phase. However, there were increased differences in the AP translation of the tibia for the three PS UKA models from the native knee during the swing phase, which reached a maximum of 1.6 mm. In particular, C-PS UKA showed less anterior translations of the tibia than the native knee and AM-PS UKA, respectively, during the swing phase. Internal rotation of the tibia was greater in normal knee than that in the three PS UKA models up to 1.4° additional internal rotation of the tibia in the 80% of the stance phase, which was predicted in the three PS UKA models during gait cycle loading condition. However, less internal rotation of the tibia was found in AM-PS UKA and C-PS UKA models than in the native knee during the swing phase. Greater anterior translation and internal rotation were found during swing phase in NC-PS UKA than native knee. The AM-PS UKA model showed the closest kinematics to the normal knee. The AMB UKA showed the most similar value to the native knee during gait cycle loading condition.

**Figure 5:**
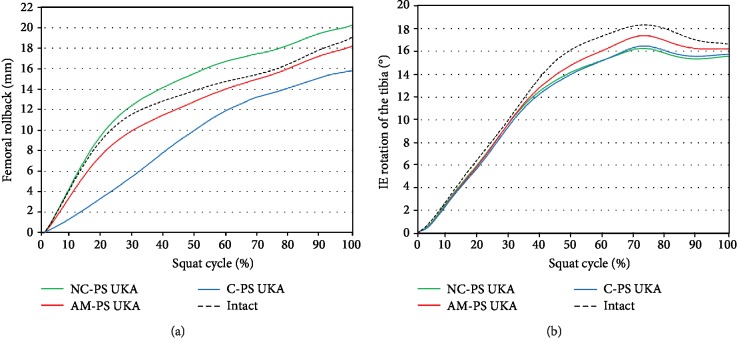
Comparison of (a) femoral rollback and (b) IE rotation of the tibia for the three types of PS UKA and natural knee model under the squat cycle.

A femoral rollback and IE rotation of the three PS UKA models and native knee under squat conditions are shown in Figure 5. The kinematics of the AM-PS UKA model was closer to normal femoral rollback and tibial internal rotation than in the other two PS UKA models. These results are similar with femoral rollback and tibial internal rotation of mobile-bearing UKA models. However, all three fixed-bearing UKA models showed less internal rotation than the native knee during a squat activity. FMB UKA showed a greater rotation under deep-knee-bend conditions than that of the natural knee.

## 4. Discussion

The most important finding of this study was that the AM-PS UKA showed the closest kinematics to the native knee. Based on the current findings, it appears that the bony geometry of the native medial femoral condyle and tibial plateau is functionally restored by the implant.

The advantage of UKA is the greater preservation of the knee joint anatomy, which helps restore the normal joint function. Gait analysis data have shown that clinically successful medial UKA leads to a similar percentage of patients (70%) having normal, biphasic quadriceps use compared to healthy patients (79%) [43]. Owing to the growing utilization of UKA, a wide range of designs have become available on the market [44–46]. However, these designs have had limited success in the preservation of normal knee feeling and function owing to their failure to incorporate the important aspects of normal knee biomechanics.

Preservation of the native knee function is the ultimate goal of advanced prosthetic knee designs. The best advantage is that PS implants for a unilateral indication will provide significantly less overhang and undercoverage, as well as superior coverage, of the cortical rim of the tibia compared to conventional implants [9]. Several previous studies have demonstrated that an overhang of the tibial baseplate can lead to significant clinical issues with pain and impingement [26, 47, 48]. It was demonstrated that, in UKA, patients with a significant overhang have an increased risk of worse postoperative knee and pain scores [47]. A previous study conducted an analysis of the theoretical designs of UKA versus known shapes of commercially available implants on 34 tibiae [26, 47, 48]. The results indicate that the theoretical design in which both the shape and size can be altered provides significantly better cortical rim coverage than commercially available implants regardless of the shape [26, 47, 48]. Therefore, PS resurfacing implants allow a femoral bone-preserving approach with enhanced cortical bone support on the tibia, overcoming the critical design limitations of conventional commercial implants [49]. In addition, PS resurfacing implants may preserve the normal anatomy and joint function, and may improve the clinical results [49].

We previously investigated the biomechanical effect between the PS and conventional UKA [18]. We proved that PS UKA provides mechanics closer to those of a normal knee joint. However, a previously used conformity design of a tibial insert in PS UKA was flat to avoid edge loading, as previously mentioned [18]. In general, a flat design is used for a fixed-bearing UKA design, and a conforming design was used for a mobile-bearing UKA design [43, 44]. Previous studies have mostly described the condylar shape of the knee implant for conformity [50–52]. These studies showed how changes in the conformity of the femoral and tibial component can impact the performance metrics [52]. However, the authors were curious regarding how a PS UKA design provides closer kinematics to a native knee with respect to tibial insert conformity. Therefore, we have three different NC, AM, and C conformity designs of a developed fixed bearing of tibial insert, which were compared with the kinematics from a native knee using a computational simulation. Based on this study, the intact knee model had undertaken a series of rigorous validation steps, the results of which show good agreement with previous experimental or FE simulations. Therefore, the UKA models developed in this study and the following analysis can be considered reasonable.

The kinematics of a normal knee has been shown to be activity dependent. During a high flexion such as a deep knee bend and sitting in a chair, the normal knee shows an overall medial pivot with a greater rollback of the lateral than the medial condyle [53, 54]. However, during low flexion activities such as stair climbing and walking, normal knees exhibit a variable pivot pattern [54, 55]. The native tibial geometry consists of a shallow medial plateau and a convex lateral side, which makes the knee anatomy asymmetric, as previously mentioned. Further, the medial and lateral menisci provide a differential medial and lateral constraint contributing to the differential AP kinematics of the medial and lateral femoral condyle through the range of motion. A previous study showed that the tibial articular surfaces of contemporary implants are in conflict with a normal knee motion and soft-tissue function [55]. Recent studies have shown that such AM implants may represent an important procedure in our attempt to preserve the normal kinematic function of the knee [54, 56]. Recently developed devices have applied such an AM design even in conventional implants [57]. Our results indicate that AM-PS UKA shows closer kinematics to a normal knee than the other PS UKA models during gait and squat loading. Such kinematic alterations in two PS UKA designs are concerned with their nonanatomic articular surface geometries.

We showed that the three different PS UKA models provide greater external rotation and similar AP translation to a native knee during the stance phase, which are similar to a previous study [58]. An interesting finding was shown in the swing phases. NC-PS TKA showed a greater anterior translation and internal rotational of the tibia than C-PS TKA during the swing phase. This is due to conformity between the femoral and tibial insert. The axial force makes up a large part of the loading during the stance phase, and thus high conforming C-PS UKA showed a medial pivot motion. There is no axial force in the swing phase because it is a passive flexion, and thus a flat design NC-PS UKA showed high kinematics owing to fewer constraints.

AM-PS UKA showed the closest native knee kinematics during squat loading similar to the gait cycle. In addition, AM-PS UKA and C-PS UKA models showed less femoral rollback than the native knee during a squat. In contrast, greater femoral rollback was found in NC-PS UKA than native knee during a squat. However, all the three PS UKA models showed less internal rotation than the native knee during a squat. A previous study showed a similar trend [59]. NC-PS UKA showed a different trend in different design PS UKA in femoral rollback. It was because of that there was no resistance to femoral rollback in flat tibial insert design. In addition, this flat tibial insert design of NC-PS UKA showed less internal rotation than two PS UKA designs having curvature during a squat. NC-PS UKA also showed a different trend in internal rotation than native knee during swing phase and squat, and this different was due to the difference in loading condition. In general, flexion is dominant and effect of tibial insert design is relatively less in swing phase, but flexion and axial forces are involved in squat so it affects relatively much in tibial insert design. The previous study showed that AMB UKA is similar to the native knee of kinematics in the gait and squat cycle among the mobile-bearing designs. FMB UKA appeared higher kinematics than CMB UKA during swing phase in the gait cycle. And CMB UKA, which has a conforming to native knee rather than FMB UKA, has similar kinematics to the native knee in the squat condition [22].

In terms of clinical relevance, tibial insert conformity should not be designed well in a PS UKA design because high conformity cannot lead to natural knee kinematics. Previous studies have shown that fixed-bearing UKA leads to poor functional results after an insertion-constrained tibial insert [60, 61]. We recommend that conformity of the tibial insert should be followed with the current anatomy in the PS UKA design.

AM-PS UKA has a moderately dished medial surface that avoids an over constraint of the medial condyle motion. The moderately dished medial compartment has an advantage in terms of laxity, accommodating variations in the pivot center during activities of limited flexion, as well as intrasubject variations in the knee kinematics [55, 62]. However, even AM-PS UKA cannot restore the native knee kinematics, the reason for which is that the stiffness changes even if AM-PS UKA has a perfectly mimetic anatomy. A change in stiffness might affect the kinematics. In addition, menisci have mobile characteristics that have not been considered. A recent *in vitro* biomechanical study showed that the following mobile-bearing UKA kinematics were close to those of the native knee, particularly during passive motion.

Two strengths of the present study should be highlighted. First, in contrast to previous UKA studies, the FE model in this study included the tibia, femur, and related soft tissues. Second, in contrast to the current biomechanical UKA model, this study included the gait cycle and squat loading, as opposed to a simple vertical static loading condition. Nevertheless, the current study has several limitations. First, the anatomy of the UKA design was based on a single subject. However, the benefit of a computational simulation with a single subject are a determination of the component design effect within the same subject and the elimination of variables, such as the weight, height, bony geometry, ligament properties, and component size [18]. Second, we validated only the initial model. However, this method has been widely used in the field of orthopedic biomechanics. Third, the results do not predict clinical results and patient satisfaction. Finally, the model assumes the material properties and attachment points of the ligaments based on highly variable values from the literature. However, our objective was not to determine the actual values for muscle and ligament forces, but instead to determine the effect of variability in PS UKA with respect to tibial insert conformity for the variables of interest.

## 5. Conclusions

How much the PS UKA design differs in terms of conformity of the kinematics from a native knee was investigated using a finite element computational simulation. The kinematics of the PS UKA models was found to be broadly similar to that of the native knee model. Our results show that the conformity of each component in PS UKA is an important factor in knee joint kinematics; however, the anatomy of a mimetic UKA design is unable to restore perfect native kinematics.

## Data Availability

The data used to support the findings of this study are included in the article and previously reported data. This prior study is cited at relevant places within the text as the reference [22].

## References

[1] Price A. J., Svard U. (2011). A second decade lifetable survival analysis of the Oxford unicompartmental knee arthroplasty. *Clinical Orthopaedics and Related Research*.

[2] Vince K. G., Cyran L. T. (2004). Unicompartmental knee arthroplasty: new indications, more complications?. *The Journal of Arthroplasty*.

[3] Cameron H. U., Jung Y. B. (1988). A comparison of unicompartmental knee replacement with total knee replacement. *Orthopaedic Review*.

[4] Furnes A., Lie S. A., Havelin L. I., Engesaeter L. B., Vollset S. E. (1996). The economic impact of failures in total hip replacement surgery: 28,997 cases from the Norwegian Arthroplasty Register, 1987-1993. *Acta Orthopaedica Scandinavica*.

[5] Berend K. R., Lombardi A. V., Mallory T. H., Adams J. B., Groseth K. L. (2005). Early failure of minimally invasive unicompartmental knee arthroplasty is associated with obesity. *Clinical Orthopaedics and Related Research*.

[6] Newman J. H., Ackroyd C. E., Shah N. A. (1998). Unicompartmental or total knee replacement?. *The Journal of Bone and Joint Surgery. British volume*.

[7] Scott R. D., Santore R. F. (1981). Unicondylar unicompartmental replacement for osteoarthritis of the knee. *The Journal of Bone & Joint Surgery*.

[8] Rand J. A., Ilstrup D. M. (1991). Survivorship analysis of total knee arthroplasty. Cumulative rates of survival of 9200 total knee arthroplasties. *The Journal of Bone & Joint Surgery*.

[9] Carpenter D. P., Holmberg R. R., Quartulli M. J., Barnes C. L. (2014). Tibial plateau coverage in UKA: a comparison of patient specific and off-the-shelf implants. *The Journal of Arthroplasty*.

[10] Mensch J. S., Amstutz H. C. (1975). Knee morphology as a guide to knee replacement. *Clinical Orthopaedics and Related Research*.

[11] Hashemi J., Chandrashekar N., Gill B. (2008). The geometry of the tibial plateau and its influence on the biomechanics of the tibiofemoral joint. *The Journal of Bone and Joint Surgery-American Volume*.

[12] Servien E., Saffarini M., Lustig S., Chomel S., Neyret P. (2008). Lateral versus medial tibial plateau: morphometric analysis and adaptability with current tibial component design. *Knee Surgery, Sports Traumatology, Arthroscopy*.

[13] Koeck F. X., Beckmann J., Luring C., Rath B., Grifka J., Basad E. (2011). Evaluation of implant position and knee alignment after patient-specific unicompartmental knee arthroplasty. *The Knee*.

[14] Steklov N., Slamin J., Srivastav S., D'Lima D. (2010). Unicompartmental knee resurfacing: enlarged tibio-femoral contact area and reduced contact stress using novel patient-derived geometries. *Open Biomedical Engineering Journal*.

[15] Freeman M. A. R., Pinskerova V. (2005). The movement of the normal tibio-femoral joint. *Journal of Biomechanics*.

[16] Fox A. J. S., Bedi A., Rodeo S. A. (2012). The basic science of human knee menisci: structure, composition, and function. *Sports Health: A Multidisciplinary Approach*.

[17] Masouros S. D., McDermott I. D., Amis A. A., Bull A. M. J. (2008). Biomechanics of the meniscus-meniscal ligament construct of the knee. *Knee Surgery, Sports Traumatology, Arthroscopy*.

[18] Kang K. T., Son J., Suh D. S., Kwon S. K., Kwon O. R., Koh Y. G. (2018). Patient-specific medial unicompartmental knee arthroplasty has a greater protective effect on articular cartilage in the lateral compartment: a finite element analysis. *Bone & Joint Research*.

[19] Kang K. T., Kim S. H., Son J., Lee Y. H., Koh Y. G. (2017). Validation of a computational knee joint model using an alignment method for the knee laxity test and computed tomography. *Bio-medical Materials and Engineering*.

[20] Kang K. T., Kwon S. K., Son J., Kwon O. R., Lee J. S., Koh Y. G. (2018). The increase in posterior tibial slope provides a positive biomechanical effect in posterior-stabilized total knee arthroplasty. *Knee Surgery, Sports Traumatology, Arthroscopy*.

[21] Koh Y. G., Park K. M., Lee H. Y., Kang K. T. (2019). Influence of tibiofemoral congruency design on the wear of patient-specific unicompartmental knee arthroplasty using finite element analysis. *Bone & Joint Research*.

[22] Koh Y. G., Lee J. A., Lee H. Y., Chun H. J., Kim H. J., Kang K. T. (2019). Anatomy-mimetic design preserves natural kinematics of knee joint in patient-specific mobile-bearing unicompartmental knee arthroplasty. *Knee Surgery, Sports Traumatology, Arthroscopy*.

[23] Harrysson O. L., Hosni Y. A., Nayfeh J. F. (2007). Custom-designed orthopedic implants evaluated using finite element analysis of patient-specific computed tomography data: femoral-component case study. *BMC Musculoskeletal Disorders*.

[24] van den Heever D. J., Scheffer C., Erasmus P., Dillon E. (2011). Contact stresses in a patient-specific unicompartmental knee replacement. *Clinical Biomechanics*.

[25] Leng L., Tan Y., Gong F. (2015). Differentiation of primordial germ cells from induced pluripotent stem cells of primary ovarian insufficiency. *Human Reproduction*.

[26] Fitzpatrick C., FitzPatrick D., Lee J., Auger D. (2007). Statistical design of unicompartmental tibial implants and comparison with current devices. *The Knee*.

[27] Harman M. K., Schmitt S., Rössing S. (2010). Polyethylene damage and deformation on fixed-bearing, non-conforming unicondylar knee replacements corresponding to progressive changes in alignment and fixation. *Clinical Biomechanics*.

[28] Piefer J. W., Pflugner T. R., Hwang M. D., Lubowitz J. H. (2012). Anterior cruciate ligament femoral footprint anatomy: systematic review of the 21st century literature. *Arthroscopy: The Journal of Arthroscopic & Related Surgery*.

[29] Bowman K. F., Sekiya J. K. (2010). Anatomy and biomechanics of the posterior cruciate ligament, medial and lateral sides of the knee. *Sports Medicine and Arthroscopy Review*.

[30] Baldwin J. L. (2009). The anatomy of the medial patellofemoral ligament.

[31] Blankevoort L., Huiskes R. (1996). Validation of a three-dimensional model of the knee. *Journal of Biomechanics*.

[32] Mesfar W., Shirazi-Adl A. (2005). Biomechanics of the knee joint in flexion under various quadriceps forces. *The Knee*.

[33] Peña E., Calvo B., Martinez M. A., Palanca D., Doblaré M. (2006). Why lateral meniscectomy is more dangerous than medial meniscectomy. A finite element study. *Journal of Orthopaedic Research*.

[34] Kwon O. R., Kang K. T., Son J., Suh D. S., Baek C., Koh Y. G. (2017). Importance of joint line preservation in unicompartmental knee arthroplasty: finite element analysis. *Journal of Orthopaedic Research : Official Publication of the Orthopaedic Research Society*.

[35] Godest A. C., Beaugonin M., Haug E., Taylor M., Gregson P. J. (2002). Simulation of a knee joint replacement during a gait cycle using explicit finite element analysis. *Journal of Biomechanics*.

[36] Inoue S., Akagi M., Asada S., Mori S., Zaima H., Hashida M. (2016). The valgus inclination of the tibial component increases the risk of medial tibial condylar fractures in unicompartmental knee arthroplasty. *The Journal of Arthroplasty*.

[37] Kang K. T., Koh Y. G., Son J. (2016). Measuring the effect of femoral malrotation on knee joint biomechanics for total knee arthroplasty using computational simulation. *Bone & Joint Research*.

[38] Kutzner I., Heinlein B., Graichen F. (2010). Loading of the knee joint during activities of daily living measured in vivo in five subjects. *Journal of Biomechanics*.

[39] Halloran J. P., Clary C. W., Maletsky L. P., Taylor M., Petrella A. J., Rullkoetter P. J. (2010). Verification of predicted knee replacement kinematics during simulated gait in the Kansas knee simulator. *Journal of Biomechanical Engineering*.

[40] Kang K. T., Koh Y. G., Jung M. (2017). The effects of posterior cruciate ligament deficiency on posterolateral corner structures under gait- and squat-loading conditions: a computational knee model. *Bone & Joint Research*.

[41] Kang K. T., Koh Y. G., Son J. (2017). Finite element analysis of the biomechanical effects of 3 posterolateral corner reconstruction techniques for the knee joint. *Arthroscopy: The Journal of Arthroscopic & Related Surgery*.

[42] Grood E. S., Suntay W. J. (1983). A joint coordinate system for the clinical description of three-dimensional motions: application to the knee. *Journal of Biomechanical Engineering*.

[43] Chassin E. P., Mikosz R. P., Andriacchi T. P., Rosenberg A. G. (1996). Functional analysis of cemented medial unicompartmental knee arthroplasty. *The Journal of Arthroplasty*.

[44] Emerson R. H., Hansborough T., Reitman R. D., Rosenfeldt W., Higgins L. L. (2002). Comparison of a mobile with a fixed-bearing unicompartmental knee implant. *Clinical Orthopaedics and Related Research*.

[45] Confalonieri N., Manzotti A., Pullen C. (2004). Comparison of a mobile with a fixed tibial bearing unicompartimental knee prosthesis: a prospective randomized trial using a dedicated outcome score. *The Knee*.

[46] Gleeson R. E., Evans R., Ackroyd C. E., Webb J., Newman J. H. (2004). Fixed or mobile bearing unicompartmental knee replacement? A comparative cohort study. *The Knee*.

[47] Chau R., Gulati A., Pandit H. (2009). Tibial component overhang following unicompartmental knee replacement—does it matter?. *The Knee*.

[48] Gudena R., Pilambaraei M. A., Werle J., Shrive N. G., Frank C. B. (2013). A safe overhang limit for unicompartmental knee arthroplasties based on medial collateral ligament strains: an in vitro study. *The Journal of Arthroplasty*.

[49] Fitz W. (2009). Unicompartmental knee arthroplasty with use of novel patient-specific resurfacing implants and personalized jigs. *The Journal of Bone and Joint Surgery-American Volume*.

[50] Fregly B. J., Marquez-Barrientos C., Banks S. A., DesJardins J. D. (2010). Increased conformity offers diminishing returns for reducing total knee replacement wear. *Journal of Biomechanical Engineering*.

[51] Sathasivam S., Walker P. S. (1999). The conflicting requirements of laxity and conformity in total knee replacement. *Journal of Biomechanics*.

[52] Ardestani M. M., Moazen M., Jin Z. (2015). Contribution of geometric design parameters to knee implant performance: conflicting impact of conformity on kinematics and contact mechanics. *The Knee*.

[53] Johal P., Williams A., Wragg P., Hunt D., Gedroyc W. (2005). Tibio-femoral movement in the living knee. A study of weight bearing and non-weight bearing knee kinematics using 'interventional' MRI. *Journal of Biomechanics*.

[54] Varadarajan K. M. M., Zumbrunn T., Rubash H. E., Malchau H., Li G., Muratoglu O. K. (2015). Cruciate retaining implant with biomimetic articular surface to reproduce activity dependent kinematics of the normal knee. *The Journal of Arthroplasty*.

[55] Li J. S., Hosseini A., Cancre L., Ryan N., Rubash H. E., Li G. (2013). Kinematic characteristics of the tibiofemoral joint during a step-up activity. *Gait & Posture*.

[56] Koh Y. G., Son J., Kwon O. R., Kwon S. K., Kang K. T. (2018). Patient-specific design for articular surface conformity to preserve normal knee mechanics in posterior stabilized total knee arthroplasty. *Bio-medical Materials and Engineering*.

[57] Anonymous (2016). *JOURNEY II Active knee solutions*.

[58] Hopkins A. R., New A. M., Rodriguez-y-Baena F., Taylor M. (2010). Finite element analysis of unicompartmental knee arthroplasty. *Medical Engineering & Physics*.

[59] Mochizuki T., Sato T., Koga Y. (2013). In vivo pre- and postoperative three-dimensional knee kinematics in unicompartmental knee arthroplasty. *Journal of Orthopaedic Science*.

[60] Hodge W. A., Chandler H. P. (1992). Unicompartmental knee replacement: a comparison of constrained and unconstrained designs. *The Journal of Bone & Joint Surgery*.

[61] Bernasek T. L., Rand J. A., Bryan R. S. (1988). Unicompartmental porous coated anatomic total knee arthroplasty. *Clinical Orthopaedics and Related Research*.

[62] Benoit D. L., Ramsey D. K., Lamontagne M., Xu L., Wretenberg P., Renström P. (2007). In vivo knee kinematics during gait reveals new rotation profiles and smaller translations. *Clinical Orthopaedics and Related Research*.

